# The gene network and knowledge base on human thermoregulation

**DOI:** 10.18699/vjgb-25-106

**Published:** 2025-12

**Authors:** E.V. Ignatieva, P.S. Demenkov, A.G. Bogomolov, R.A. Ivanov, S.A. Lashin, A.D. Mikhailova, A.E. Alekseeva, N.S. Yudin

**Affiliations:** Institute of Cytology and Genetics of the Siberian Branch of the Russian Academy of Sciences, Novosibirsk, Russia; Institute of Cytology and Genetics of the Siberian Branch of the Russian Academy of Sciences, Novosibirsk, Russia; Institute of Cytology and Genetics of the Siberian Branch of the Russian Academy of Sciences, Novosibirsk, Russia; Institute of Cytology and Genetics of the Siberian Branch of the Russian Academy of Sciences, Novosibirsk, Russia; Institute of Cytology and Genetics of the Siberian Branch of the Russian Academy of Sciences, Novosibirsk, Russia Novosibirsk State University, Novosibirsk, Russia; Novosibirsk State University, Novosibirsk, Russia; Novosibirsk State University, Novosibirsk, Russia; Institute of Cytology and Genetics of the Siberian Branch of the Russian Academy of Sciences, Novosibirsk, Russia

**Keywords:** heat, cold, gene network, database, microRNA, evolution, phylostratigraphy, gene age, тепло, холод, генная сеть, база данных, микроРНК, эволюция, филостратиграфия, возраст гена

## Abstract

Reconstruction and analysis of gene networks regulating biological processes are among the modern methodological approaches for studying complex biological systems that ensure the vital activity of organisms. Thermoregulation is an important evolutionary acquisition of warm-blooded animals. Multiple physiological systems (nervous, cardiovascular, endocrine, respiratory, muscular, etc.) are involved in this process, maintaining stable body temperature despite changes in ambient temperature. This study aims to perform a computer reconstruction of the human thermoregulation gene network and present the results in the Termo_Reg_Human 1.0 knowledge base. The gene network was reconstructed using the ANDSystem software and information system, designed for the automated extraction of knowledge and facts from scientific publications and biomedical databases based on machine learning and artificial intelligence methods. The Termo_Reg_Human 1.0 knowledge base (https://www.sysbio.ru/ThermoReg_Human/) contains information about the human thermoregulation gene network, including a description of 469 genes, 473 proteins, and 265 microRNAs important for its functioning, interactions between these objects, and the evolutionary characteristics of the genes. Using the ANDVisio software tool (a module of ANDSystem), each gene, protein, and microRNA involved in the thermoregulation of the human body was prioritized according to its functional significance, i. e., the number of interactions with other objects in the reconstructed gene network. It was found that the key objects with the largest number of functional interactions in the human thermoregulation gene network included the UCP1, VEGFA, PPARG and DDIT3 genes; STAT3, JUN, VEGFA, TLR4 and TNFA proteins; and the microRNAs hsa-mir-335 and hsa-mir-26b. We revealed that the set of 469 human genes from the network was enriched with genes whose ancestral forms originated at an early evolutionary stage (Unicellular organisms, the root of the phylostratigraphic tree) and at the stage of Vertebrata divergence.

## Abstract (ru)

Реконструкция и анализ генных сетей, регулирующих биологические процессы, – один из эффективных подходов к исследованию сложных систем обеспечения жизнедеятельности организмов. Терморегуляция – важное эволюционное приобретение человека и других теплокровных животных. Терморегуляция осуществляется при участии многих физиологических систем организма (нервной, сердечно-сосудистой, эндокринной, дыхательной, мышечной и т. д.), что способствует поддержанию относительно постоянной температуры тела в условиях колебания температуры окружающей среды. Цель работы – компьютерная реконструкция генной сети терморегуляции человека и представление полученных результатов в соответствующей базе знаний Termo_Reg_Human 1.0. Генная сеть реконструирована с использованием программно-информационной системы ANDSystem, предназначенной для автоматизированного извлечения знаний и фактов из текстов научных публикаций и баз данных биомедицинской направленности, основанной на методах машинного обучения и искусственного интеллекта. База знаний Termo_ Reg_Human 1.0 (https://www.sysbio.ru/ThermoReg_Human/) содержит информацию о генной сети терморегуляции человека, включая описание 469 генов, 473 белков и 265 микроРНК, значимых для ее функционирования; взаимодействиях между этими объектами, а также эволюционные характеристики генов. С использованием программного инструмента ANDVisio (модуля системы ANDSystem) проведена приоритизация каждого гена, белка и микроРНК, участвующих в терморегуляции организма человека по их функциональной нагруженности – количеству связей с другими объектами реконструированной генной сети. Установлено, что к числу ключевых объектов, имеющих наибольшее количество функциональных связей в генной сети терморегуляции человека, относятся гены UCP1, VEGFA, PPARG, DDIT3, белки STAT3, JUN, VEGFA, TLR4, TNFA и микроРНК hsa-mir-335 и hsa-mir-26b. Обнаружено обогащение генной сети терморегуляции генами, предковые варианты которых сформировались на эволюционных этапах появления одноклеточных организмов и дивергенции позвоночных

## Introduction

Humans and most other mammals are homoiothermic, capable of maintaining a relatively constant body temperature when the ambient temperature varies (Osvath et al., 2024). Human thermoregulation is carried out with the participation of: 1) thermoreceptors located on the body’s surface and in the internal organs; 2) afferent neural signal transmission pathways; 3) thermoregulatory centers in the hypothalamus and other parts of the brain; 4) efferent neural pathways that control adaptive reactions (Nakamura, 2024). Such adaptive reactions include: a) shivering and nonshivering thermogenesis (chemical mechanisms of thermoregulation) (Ikeda, Yamada, 2020; Dumont et al., 2025); b) physical thermoregulation, including the regulation of heat transfer through evaporation and convection, as well as thermal insulation (Nakamura, 2011; Tattersall et al., 2012); c) behavioral reactions: avoidance of open areas of the Earth’s surface characterized by extreme temperatures; crowding of individuals, etc. (Tattersall et al., 2012; Tansey, Johnson, 2015; McCafferty et al., 2017).

Chemical thermoregulation is carried out through heat production during skeletal muscle contractions (Blondin et al., 2019; Dumont et al., 2025), and nonshivering thermogenesis in brown adipose tissue (Tansey, Johnson, 2015; Ikeda, Yamada, 2020) and muscles (Blondin et al., 2019). Physical thermoregulation is carried out by changing the heat transfer from the body: conduction, radiation, perspiration, evaporation of water from the respiratory passages, thermal insulation due to the subcutaneous fat layer, piloerection (Nakamura, 2011; Tattersall et al., 2012). Both chemical and physical thermoregulatory processes are actively controlled by the neuroendocrine system (Charkoudian et al., 2017; Nakamura, 2024; Mittag, Kolms, 2025).

In addition, the thermoregulatory reactions are associated with changes in the cardiovascular system (Tansey, Johnson, 2015). Thus, thermoregulation is provided by a variety of biological processes occurring in the nervous, endocrine, cardiovascular, respiratory, muscular and other body systems. The genetic regulatory mechanisms controlling the above processes also play a significant role in thermoregulation (Festuccia et al., 2009; Rehman et al., 2013; Li et al., 2015; Horii et al., 2019; Xiao et al., 2019; Kudsi et al., 2022; Song et al., 2022; Valdivia et al., 2023).

Reconstruction and analysis of gene networks regulating biological processes are among the effective approaches to study complex biological systems that ensure vital activity of organisms (Ignatieva et al., 2017; Saik et al., 2018; Mustafin et al., 2019, 2021; Mikhailova et al., 2024). A large amount of experimental genetic data has been accumulated on the problem of thermoregulation, presented in tens of thousands of scientific publications and many specialized databases (e. g. KEGG Pathway, WikiPathways, MetaCyc, REACTOME, etc.). In this regard, in our work, we reconstructed the human thermoregulation gene network using the ANDSystem software and information system, designed for the automated extraction of knowledge and facts from the texts of scientific publications and biomedical databases using machine learning and artificial intelligence methods (Ivanisenko V.A. et al., 2019; Ivanisenko T.V. et al., 2024). The results obtained from the analysis of 30 million publications are accumulated in the specialized knowledge base of the ANDSystem in the form of a global knowledge graph (Ivanisenko T.V., 2024).

Information on the reconstructed human thermoregulatory gene network is presented in the Termo_Reg_Human 1.0. knowledge base (https://www.sysbio.ru/ThermoReg_ Human/), including descriptions of 469 genes, 473 proteins and 265 microRNAs important for gene network functioning, as well as interactions between them

Each gene, protein, and microRNA involved in human body thermoregulation was prioritized according to their functional load, i. e., the number of interactions with other objects of the reconstructed gene network, using the ANDVisio software tool (a module of the ANDSystem). The key objects with the largest number of functional interactions in the human thermoregulation gene network were found: the UCP1, VEGFA, PPARG and DDIT3 genes, the STAT3, JUN, VEGFA, TLR4 and TNFA proteins, and microRNAs hsa-mir-335 and hsa-mir-26b.

The Termo_Reg_Human 1.0 knowledge base also presents the results of an evolutionary analysis of genes functioning in the thermoregulation gene network: this gene network was enriched with genes, the ancestral forms of which emerged at two important evolutionary stages corresponding to a) the appearance of unicellular organisms and b) the divergence of vertebrates

## Materials and methods

**Lists of genes used for building a gene network. **The list of human genes involved in thermoregulation was compiled based on the Gene Ontology, EntrezGene, and ANDSystem databases (Ivanisenko V.A. et al., 2019) using the keywords shown in Supplementary Material S11.


Supplementary Materials are available in the online version of the paper: https://vavilov.elpub.ru/jour/manager/files/Suppl_Ignatieva_Engl_29_7.pdf


**Building of the gene network.** The gene network of thermoregulation was built using the ANDSystem software and information system (Ivanisenko V.A. et al., 2019; Ivanisenko T.V. et al., 2024). ANDSystem, based on machine learning and artificial intelligence methods, is designed for the automated extraction of knowledge and facts about the structural and functional organization of gene networks from scientific publications and biomedical factographical databases. The information obtained in this way is accumulated in the specialized knowledge base of ANDSystem in the form of a global knowledge graph (Ivanisenko T.V. et al., 2024). Based on this information, a reconstruction of the graphs of target gene networks is carried out, the nodes of which correspond to molecular genetic objects (genes, RNA, proteins and metabolites), functioning as part of gene networks, and the edges connecting these nodes indicate the functional interactions between objects. Supplementary Material S2 provides a detailed description of the reconstruction process of the human thermoregulatory gene network.

**Prioritization of genes, proteins, and microRNAs according to their functional significance in the human thermoregulation gene network. **Prioritization of gene network nodes (genes, microRNAs and proteins) was performed using the ANDVisio software tool (a module of the ANDSystem). The number of interactions with other objects was calculated for a specific object in the human thermoregulation gene network graph. Next, the probability of obtaining the observed number of interactions for random reasons was estimated for each gene network object. Next, the probability of observing this number of interactions involving this specific object of the gene network by chance was estimated. The probability was calculated using a hypergeometric test:

**Formula. 1.**
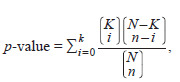
Formula 1

where: k – the number of interactions of this specific object (node) in the gene network; n – the number of objects (nodes) involved in the gene network under consideration; K – the number of interactions of this specific object (node) in the ANDSystem knowledge base global network graph; N – the total number of objects (nodes) in the ANDSystem knowledge base global graph (Ivanisenko V.A. et al., 2019).

When calculating the p-value, only objects of the same type (genes, proteins, microRNA) as the considered object of the human thermoregulation gene network were taken into account. Next, correction for multiple hypothesis testing was applied (Benjamini, Yekutieli, 2001), resulting in a P-adjusted value

Analysis of the evolutionary characteristics of the genes. The analysis of the evolutionary characteristics of genes involved in the reconstructed gene network was carried out using the Orthoweb system (Ivanov et al., 2024), which calculates the phylostratigraphic index (PAI) of each gene, 1 Supplementary Materials S1–S7 are available at: https://vavilov.elpub.ru/jour/manager/files/Suppl_Ignatieva_Engl_29_7.pdf characterizing the evolutionary age of the gene. Details of the calculation procedure for the PAI index are described in Supplementary Material S2.

**Functional annotation of genes. **The identification of Gene Ontology terms associated with genes of a certain phylostratigraphic age was carried out using the DAVID web server and its GOTERM_BP_DIRECT dictionary (Sherman et al., 2022).

**Implementation of the knowledge base on human thermoregulation.** Data for the knowledge base information tables were extracted from text outputs of the ANDVisio program (a module of the ANDSystem) using original Python scripts. The online implementation of the knowledge base was performed using MySQL 5.1.73 and PHP 5.3.3. Apache HTTP Server 2.2.15 and Nginx 1.4.1 were used.

## Results and discussion

**Genes associated with thermoregulatory processes**

The search through the Gene Ontology, EntrezGene, and ANDCell (the information component of ANDSystem) databases identified 467 protein-coding genes associated with thermoregulation, as well as two genes encoding microRNAs

**The gene network of human thermoregulation**

**Fig. 1.**
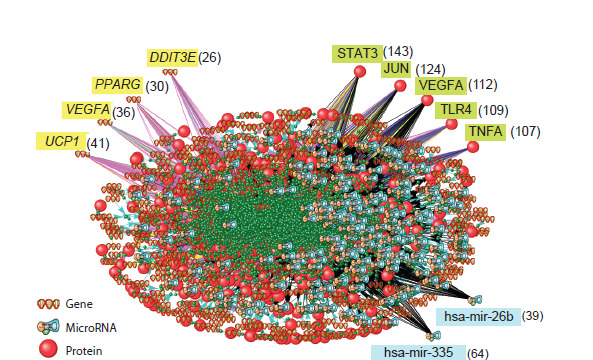
The view of the entire gene network of human thermoregulation reconstructed using the ANDSystem tool. The gene network includes 469 genes, 473 proteins, 265 microRNAs, and 7,018 interactions between these objects. Genes, proteins, and microRNAs with the highest number of interactions in the network are shown separately. Numbers in parentheses indicate the number of interactions in the network.

Based on the list of human genes involved in thermoregulation mentioned above, the gene network of human thermoregulation was reconstructed using ANDSystem. The view of the entire reconstructed gene network is shown in Figure 1. The gene network includes 469 genes, 473 proteins, 265 microRNAs and 7,018 interactions between them. The number of proteins exceeds the number of genes because the gene network contains six genes that encode more than one protein due to alternative splicing or proteolytic cleavage of the precursor protein.

It should be noted that ANDSystem identifies two types of relationships between gene networks objects, based on the analysis of scientific literature and biomedical databases: direct molecular genetic interactions between gene network objects and indirect actions, i. e. relationships in which the effect of one gene network object on another is shown, but the molecular genetic mechanism of such effect remains unknown and/or may involve intermediate objects.

**Fig. 2.**
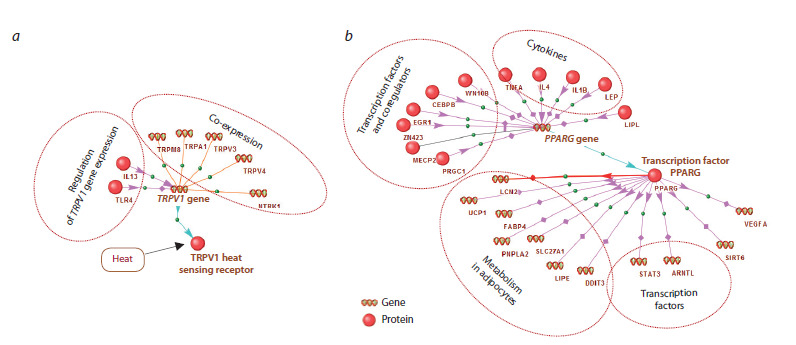
The fragments of the thermoregulation gene network shown in Figure 1. a – regulatory interactions involving the gene encoding the TRPV1 heat sensing receptor; b – regulatory interactions involving the PPARG gene and the encoded transcription factor PPARG.

Figure 2 shows two fragments of the thermoregulatory gene network. Figure 2a illustrates molecular genetic interactions of the gene encoding the thermoreceptor TRPV1, which is activated when temperature increases. According to the ANDSystem knowledge base, TRPV1 expression is regulated by interleukin 13 (IL13) and toll-like receptor 4 (TLR4). These regulatory relations are described in the articles (Rehman et al., 2015; Li et al., 2015) and can be categorized as “indirect”, since we are talking about the action of the cytokine IL13 (an extracellular signaling molecule) and the TLR4 receptor located on the cell membrane, which affect TRPV1 expression through signal transduction pathways. In addition, TRPV1 is coexpressed with other genes from the thermoregulation gene network, including thermoreceptor-encoding genes (TRPM8, TRPA1, TRPV3, TRPV4), as well as NTRK1 encoding neurotrophic receptor tyrosine kinase 1. The experiments that revealed the coexpression of these genes are described in the research papers (Zhu, Oxford, 2007; Cao et al., 2009; Cheng et al., 2011; Gouin et al., 2012; Nguyen et al., 2017).

Figure 2b shows the regulatory relationships involving the PPARG gene and its encoded protein. PPARG expression is regulated by transcription factors ZN423, EGR1, CEBPB, which affect the level of transcription by interacting with DNA in the PPARG regulatory regions. PPARG expression is also regulated by transcription cofactors MECP2 and PRGC1/ PGC-1-alpha and the WN10B protein, which activates the Wnt signaling cascade. In addition, cytokines TNF, IL4, IL1B, and LEP are involved in the regulation of PPARG expression. The transcription factor PPARG, encoded by the gene under consideration, controls the transcription of a) genes regulating metabolic processes in adipocytes: LCN2, UCP1, FABP4, PNPLA2, SLC27A1, LIPE, and DDIT3; b) genes encoding transcription factors STAT3 and ARNTL; and c) the SIRT6 gene encoding the NAD-dependent protein deacetylase. The references to scientific publications supporting these interactions are provided in Supplementary Material S3.

**The Termo_Reg_Human knowledge base**

At the next stage of the study, the Termo_Reg_Human 1.0. knowledge base (https://www.sysbio.ru/ThermoReg_Human/) was developed. This knowledge base contains data on 469 genes, 473 proteins, and 265 microRNAs involved in human thermoregulation

**Fig. 3.**
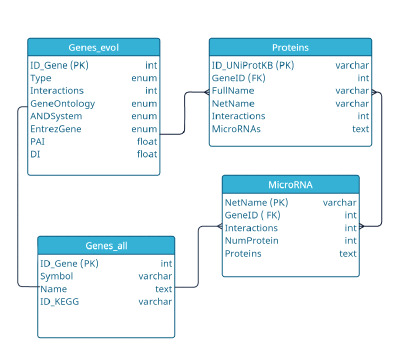
Structure of the Termo_Reg_Human 1.0. knowledge base

Termo_Reg_Human 1.0. contains four main tables: *Genes_ evol, Proteins, MicroRNA *и *Genes_all *(the knowledge base scheme is shown in Figure 3).

The *Genes_evol *table contains a description of each of the 469 genes functioning as part of the human thermoregulation gene network, including: the EntrezGene GeneID, the number of interactions of the gene with other genes and proteins of the gene network, and the evidence type supporting the association of the gene with thermoregulation (Gene Ontology, ANDSystem, Entrez Gene). This table also presents such evolutionary characteristics for each protein-coding gene as the phylostratigraphic age index (PAI) and the divergence index (DI), calculated using the OrthoWeb software package (Ivanov et al., 2024).

The *Proteins *table contains data on proteins encoded by genes from the Genes_evol table. The description of each protein includes the UniProtKb Entry Name, the NCBI GeneID of the gene encoding the protein, the number of interactions the protein has in the gene network, and the names of the microRNAs that regulate protein expression.

The MicroRNA table contains information about microRNAs that regulate the expression of proteins involved in the network. These are two microRNAs encoded by genes from the list of 469 genes mentioned above, as well as additional microRNAs found using the ANDVisio program during the reconstruction of the network. The MicroRNA table shows for each microRNA: 1) microRNA name within the network; 2) official symbol of the gene encoding this microRNA; 3) the number of interactions involving this microRNA; 4) the names of proteins for which this microRNA acts as an expression regulator.

The fourth table, Genes_all, contains additional data on all 469 genes characterized in the Genes_evol table, as well as data on the genes encoding microRNAs included in the network using the ANDVisio program.

The web interface allows to view data on genes and proteins associated with thermoregulation, as well as to search for genes/proteins by identifiers or their names. In addition, a search for objects (genes, proteins, microRNAs) by the number of functional interactions in the network is available. The interface displays objects with a number of interactions exceeding the value specified by the user.

**Using data from the Termo_Reg_Human 1.0 knowledge base in bioinformatics research**

**Fig. 4.**
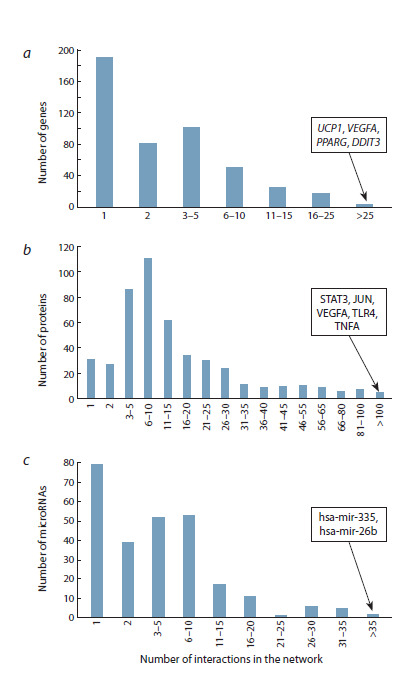
Distribution of genes, proteins, and microRNAs involved in the thermoregulatory gene network according to the number of interactions in this network (based on information from the Termo_Reg_Human 1.0 knowledge base). a – distribution of genes according to the number of interactions; b – distribution of proteins according to the number of interactions; c – distribution of microRNAs according to the number of interactions. The rectangular panels show the names of the genes, proteins, and microRNAs with the highest number of interactions

**Prioritization of genes by the number of interactions in the gene network.** Figure 4a shows the distribution of genes by the number of interactions with other objects of the human thermoregulation gene network (genes, proteins, and microRNAs). Most genes (373 out of 467) have a low number of interactions with other objects in the network (five or less). One fifth of all genes, that is, 90 genes, have from 6 to 25 interactions. Only four genes had more than 25 interactions: UCP1 (41 interactions), VEGFA (36), PPARG (30), and DDIT3 (26). A statistical analysis using the hypergeometric distribution confirmed that these four genes have significantly more interactions than would be expected by chance: the P-adjusted value varies from 2.44·10–05 for the DDIT3 gene to 1.20·10–28 for the UCP1 gene. Functional characteristics of these genes with the largest number of interactions are shown in Table 1.

**Table 1.**
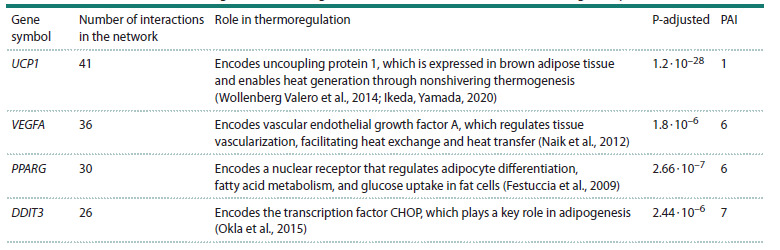
Functional characteristics of genes with the highest number of interactions in the thermoregulatory network Notе. Genes are listed in descending order based on the number of interactions in the gene network. Here and in Tables 2 and 3: P-adjusted indicates the probability of observing a given number of interactions in a network by chance, calculated using hypergeometric distribution with correction for multiple comparisons.

The UCP1 gene encodes the uncoupling protein 1 (called thermogenin), which is involved in one of the key processes of heat generation – nonshivering thermogenesis in brown adipose tissue (Wollenberg Valero et al., 2014). This protein, localized in the mitochondrial inner membrane, increases its permeability, dissipating the proton gradient generated in oxidative phosphorylation. As a result, the processes of oxidative phosphorylation and ATP synthesis are uncoupled, and heat is released (Ikeda, Yamada, 2020).The VEGFA gene encodes vascular endothelial growth factor A (Naik et al., 2012). The resulting activation of the blood supply to tissues is important for thermoregulatory processes: heat exchange between the internal parts of the body and its surface, heat dissipation through evaporation and convection, etc. (Tansey, Johnson, 2015).The PPARG gene encodes the transcription factor PPARG, which belongs to the nuclear receptor superfamily. PPARG controls the activity of genes governing the metabolism of fatty acids and glucose (Festuccia et al., 2009), and also activates the production of the UCP1 (uncoupling protein 1, thermogenin) in brown and beige adipocytes (Valdivia et al., 2023).

The DDIT3 gene encodes CHOP (C/EBP homologous protein), a transcription factor from the C/EBP family regulating differentiation of adipocyte precursor cells into mature adipocytes, which play a crucial role in nonshivering thermogenesis (Okla et al., 2015).

**Prioritization of proteins by the number of interactions in the gene network of thermoregulation.** Analysis of the thermoregulation gene network revealed that proteins generally have more interactions than genes (Fig. 4b): the proportion of proteins that had no more than five interactions was less than half of their total number (144 out of 473). 55 % of the proteins (261 proteins) had from 6 to 30 interactions, 13 % of the proteins (63 proteins) had from 31 to 100 interactions. Five proteins (STAT3, JUN, VEGFA, TLR4, TNFA) had more than 100 interactions with other network objects. A statistical analysis using the hypergeometric distribution confirmed that these five proteins have a significantly greater number of interactions with the rest of the network objects than would be expected by chance: P-adjusted value ranged from 2.04·10–18 for the TLR4 protein to 3.79·10–43 for the STAT3 protein. The characteristics of these five proteins are given in Table 2.

**Table 2.**
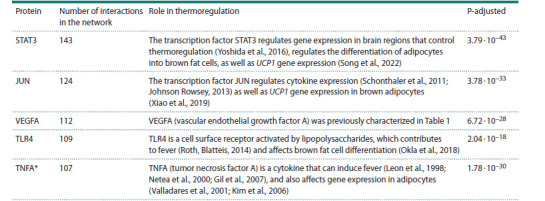
Functional characteristics of proteins with the highest number of interactions in the network of thermoregulation Notе. Proteins are listed in descending order of the number of interactions in the gene network. * TNFA is encoded by the TNF gene

STAT3 (143 interactions in the network) is a transcription factor acting at the final step of the JAK/STAT3 signal transduction pathway. STAT3 regulates adipocyte differentiation during the induction phase, and subsequent inactivation of the JAK/STAT3 pathway in these cells provides UCP1 gene expression activation and the conversion of preadipocytes into mature brown fat cells (Song et al., 2022). In addition, STAT3 is involved in the signaling pathway activated by the heat sensing receptor TRPV1 in brain regions that control body temperature (Yoshida et al., 2016).

The JUN protein (124 interactions in the network) is a subunit of the transcription factor AP1 (the JUN/FOS heterodimer). JUN is involved in the regulation of cytokine expression, thereby controlling the inflammatory processes that are associated with elevated body temperature (Schonthaler et al., 2011; Johnson Rowsey, 2013). It has been shown that when the expression of the JUN gene in the liver is inactivated in liver-specific c-Jun knock-out mice, an increase in body temperature occurs due to the activation of the sympathetic nervous system and subsequent stimulation of UCP1 expression in brown fat (Xiao et al., 2019).

As mentioned above, the VEGFA protein, which has 112 interactions in the network, controls vascular endothelium growth (Naik et al., 2012), which is important for heat exchange between tissues and the external environment (Tansey, Johnson, 2015).

TLR4 (109 interactions in the network) is a transmembrane protein, toll-like receptor 4. It can be activated by lipopolysaccharides (LPS) found in bacterial cell walls, leading to an increase in body temperature in response to infection (Roth, Blatteis, 2014). Additionally, activation of the TLR4 receptor by lipopolysaccharides leads to oxidative stress, mitochondrial dysfunction, and inhibition of the brown adipocyte differentiation (Okla et al., 2018).

The TNFA protein, tumor necrosis factor, belongs to the cytokine family (107 interactions in the network). It activates, in particular, prostaglandin synthesis in endothelial cells. These prostaglandins act on neurons in the preoptic area of the hypothalamus, the brain’s thermoregulatory center, leading to increased body temperature (Leon et al., 1998; Netea et al., 2000; Gil et al., 2007; Nakamura, 2024). TNFA has also been shown to have a direct effect on adipocytes in vitro, reducing the expression of thermogenin (UCP-1) (Valladares et al., 2001) and the enzyme triglyceride lipase ATGL/PNPLA2 (Kim et al., 2006). Thus, the cytokine TNFA plays an important role in thermoregulation, but its effect on body temperature depends on the type of cells affected by this cytokine

**Prioritization of microRNAs by the number of interactions in the gene network of thermoregulation.** MicroRNAs regulate gene expression at the translational level. These RNAs bind to the mRNA targets within miRISC complex, inhibiting protein synthesis with or without transcript degradation (O’Brien et al., 2018). According to the Termo_Reg_ Human 1.0 knowledge base, the thermoregulation gene network includes 265 microRNAs that are involved in regulating the expression of 297 genes. Data on these regulatory relationships was obtained from the miRTarBase, which contains experimentally confirmed information about interactions between microRNAs and their mRNA targets (Cui et al., 2025). The proportion of microRNAs having not more than five regulatory interactions in the network was 64 % (170 out of 265) (Fig. 4c). 35 % of the total set of microRNAs (93 out of 265) had from 6 to 30 interactions. Two microRNAs had the highest number of interactions (more than 35). These are hsa-mir-335 (64 interactions) and hsa-mir-26b (39 interactions). An assessment of the statistical significance of the number of interactions between these microRNAs and other objects of the network using the ANDVisio program showed that microRNAs hsa-mir-335 and hsa-mir-26b regulate the expression of a significantly larger number of genes from the thermoregulatory network than would be expected by chance (P-adjusted < 0.01).

The two microRNAs mentioned above are important for thermoregulatory processes (Table 3). So, hsa-mir-335 regulates the expression of thermoreceptors TRPM8 and TRPV4, as well as the VEGFA protein, one of the key proteins for thermoregulation, which is involved in 112 interactions in the network. The hsa-mir-26b microRNA regulates the expression of JUN (Jun proto-oncogene, AP-1 transcription factor subunit), which is involved in 124 interactions in the network. As noted above, JUN affects the expression of thermogenin (uncoupling protein 1, UCP1) in brown fat cells (Xiao et al., 2019). This microRNA also regulates the expression of the EDN2 (endothelin-2) protein, which controls vasoconstriction, a process that mediates physical thermoregulation (Inoue et al., 1989)

The list of genes associated with thermoregulation we have created contains the MIR21 and MIRLET7c genes. The microRNAs encoded by these genes, hsa-mir-21 and hsa-let-7c, regulate cellular processes in response to elevated temperature (Jiang et al., 2016; Permenter et al., 2019). The effect of the hsa-mir-21 and hsa-let-7c microRNAs on the expression of 15 and 5 proteins, respectively, was revealed in the reconstructed gene network (Table 3).

Among the proteins, the expression of which is regulated by hsa-mir-21, VEGFA (vascular endothelial growth factor A) was found to have 112 interactions in the network (Table 3). Multiple mentions of this protein in this report are an evidence of its important role in thermoregulation. Among the proteins, the expression of which is controlled by hsa-let-7c, the following were identified: a) COX2, a subunit of cytochrome c oxidase, involved in mitochondrial electron transport, encoded by the MT-CO2 gene (Aich et al., 2018); b) DICER1, ribonuclease type III, involved in microRNA biogenesis (Wingo et al., 2015); c) CNOT3/NOT, CCR4-NOT transcription complex subunit 3, participating in microRNA-mediated mRNA degradation (Wakiyama, Takimoto, 2022).

**Table 3.**
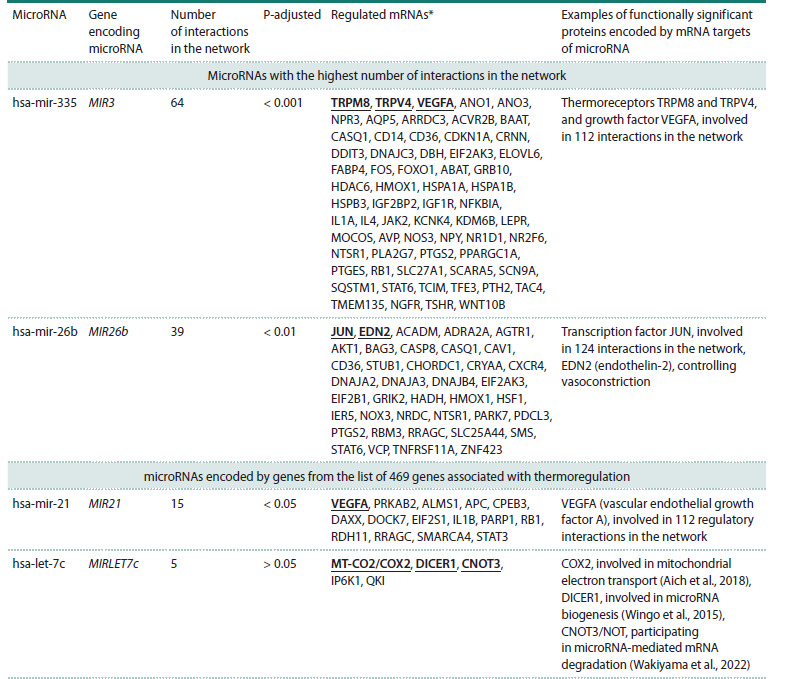
Characteristics of microRNAs with the highest functional significance within the network of human thermoregulation * mRNAs the translation of which is regulated by this microRNA (mRNAs encoding proteins described in the right column are underlined).

**Fig. 5.**
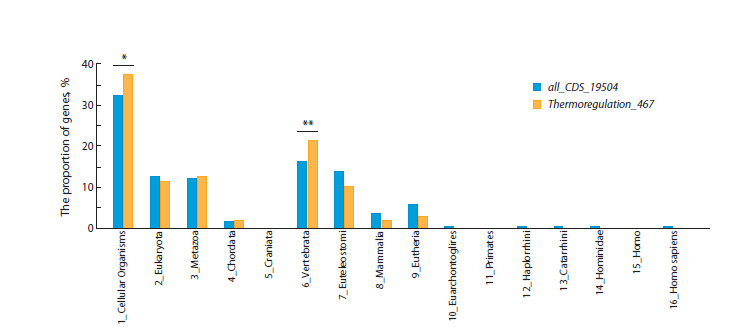
Distribution of PAI values for protein-coding genes associated with thermoregulation (Thermoregulation_467 set) and for all human protein-coding genes (all_CDS_19504 set). One asterisk (*) indicates a significant (p < 0.05) excess of the observed number of genes associated with thermoregulation corresponding to PAI = 1 (unicellular organisms, the root of the phylostratigraphic tree) over the expected number of genes with PAI = 1 calculated based on the distribution of PAI values for the complete set of protein-coding genes (all_CDS_19504 set). Two asterisks (**) show a significant (p < 0.01) excess of the observed number of genes associated with thermoregulation corresponding to PAI = 6 (the stage of Vertebrata divergence) over their expected number

**Phylostratigraphic age of genes involved in the gene network of human thermoregulation (PAI-based analysis).** The analysis of the evolutionary age of genes was carried out using the PAI (phylostratigraphic age index), the data on which were obtained from the Genes_evol information table from the Termo_Reg_Human 1.0 knowledge base. The phylostratigraphic age index was calculated using the Orthoweb system (Ivanov et al., 2024) as proposed in our previous studies (Mustafin et al., 2017). We constructed a distribution of PAI values for 467 protein-coding genes functioning in the thermoregulation gene network described in the Termo_Reg_ Human 1.0 knowledge base (the Thermoregulation_467 gene set, in Figure 5 this distribution is marked with orange bars). It turned out that this distribution has two maxima. The first of them is observed at PAI = 1 (176 genes, 38 % of their total list). The phylostratigraphic index PAI = 1 corresponds to the evolutionary stage of the emergence of unicellular organisms. The second peak is observed at PAI = 6 (100 genes associated with thermoregulation, 22 % of their total list). The phylostratigraphic index PAI = 6 corresponds to the evolutionary stage of the Vertebrata divergence.

To evaluate the statistical significance of the two peaks, a reference PAI index distribution was constructed for all human protein-coding genes (19,504 genes, the all_CDS_19504 gene set, marked in blue in Figure 5), as it was done in our previous study (Mikhailova et al., 2024). This distribution also has two, but less noticeable, peaks. Using the chi-square method, the number of genes from the Thermoregulation_467 gene set falling into peaks 1 and 6 was compared with the number of genes expected for random reasons in these peaks. In both cases, a difference was found between the observed and expected number with the level of significance p < 0.05 and p < 0.01 (Supplementary Materials S4 and S5). Thus, it was shown that the gene network of thermoregulation was enriched with genes, the ancestral forms of which originated at the early evolutionary stage (emergence of unicellular organisms, the root of the phylostratigraphic tree) and at the stage of Vertebrata divergence.

Functional analysis of the genes from the Thermoregulation_ 467 set performed using the DAVID tool showed that a group of genes with PAI = 1 is enriched with associations with the Gene Ontology terms related to transcription regulation (Supplementary Material S6), the most important mechanism for regulating gene expression in unicellular organisms. As for the group of genes with an index value of PAI = 6, it is enriched with genes involved in signal transduction (Supplementary Material S7), a vital process that ensures intercellular communications in a multicellular organism. This result is consistent with the idea that the interactions of a great number of physiological systems of the body (respiratory, circulatory, muscular, nervous, etc.) play a crucial role in the thermoregulation of the human body (Tansey, Johnson, 2015; Nakamura, 2024). In this case, the process of transcription provides genetic control over cell differentiation and formation of tissues involved in thermoregulation, and the coordination of the activity of physiological systems that ensure thermoregulation is carried out at the cellular level through signal transduction pathways.

## Conclusion

In this study, a gene network comprising human genes, microRNAs, and proteins associated with thermoregulation was built. Additionally, the Termo_Reg_Human 1.0 knowledge base was developed to systematize current data on the molecular and genetic mechanisms underlying thermoregulatory processes. Based on data contained in the knowledge base, the prioritization of genes, proteins and microRNAs by the number of interactions in the network of thermoregulation was carried out, and the evolutionary characteristics of the genes were identified.

Enrichment of the thermoregulation gene network with genes, the ancestors of which were formed at the evolutionary stages of unicellular organisms and Vertebrata divergence, was revealed. The patterns in the evolution of the genes we discovered should be taken into account when developing new concepts for the emergence of endothermy across different animal taxa (Osvath et al., 2024).

## Conflict of interest

The authors declare no conflict of interest.
